# Radius Constants for Analytic Functions with Fixed Second Coefficient

**DOI:** 10.1155/2014/898614

**Published:** 2014-07-01

**Authors:** Mahnaz M. Nargesi, Rosihan M. Ali, V. Ravichandran

**Affiliations:** ^1^Department of Mathematics, College of Natural Sciences & Mathematics, California State University, 800 North State College Boulevard, Fullerton, CA 92831-3599, USA; ^2^School of Mathematical Sciences, Universiti Sains Malaysia, 11800 Penang, Malaysia; ^3^Department of Mathematics, University of Delhi, Delhi 110 007, India

## Abstract

Let *f*(*z*) = *z* + ∑_*n*=2_^*∞*^ *a*_*n*_ *z* ^*n*^ be analytic in the unit disk with the second coefficient *a*_2_ satisfying |*a*_2_ | = 2*b*, 0 ≤ *b* ≤ 1. Sharp radius of Janowski starlikeness is obtained for functions *f* whose *n*th coefficient satisfies |*a*_*n*_ | ≤ *cn* + *d*  (*c*, *d* ≥ 0) or |*a*_*n*_ | ≤ *c*/*n*  (*c* > 0  and  *n* ≥ 3). Other radius constants are also obtained for these functions, and connections with earlier results are made.

## 1. Introduction

Let *A* denote the class of analytic functions *f* defined in the open unit disk *D*≔{*z* ∈ *C* : |*z* | <1}, normalized by *f*(0) = 0 = *f*′(0) − 1, and let *S* denote its subclass consisting of univalent functions. If *f*(*z*) = *z* + ∑_*n*=2_^*∞*^ *a*_*n*_ *z* ^*n*^ ∈ *S*, de Branges [1] obtained the sharp coefficient bound that |*a*_*n*_ | ≤*n*  (*n* ≥ 2). However, the inequality |*a*_*n*_ | ≤*n*, *n* ≥ 2, is not sufficient for *f* to be univalent; for example, *f*(*z*) = *z* + 2*z* ^2^ is clearly not a member of *S*.

Several subclasses of *S* possess a similar coefficient bound. For instance, the *n*th coefficients of starlike functions, convex functions in the direction of imaginary axis, and close-to-convex functions satisfy |*a*_*n*_ | ≤*n*  (*n* ≥ 2) [2–4]. Other examples include functions which are convex, starlike of order 1/2, and starlike with respect to symmetric points. The *n*th coefficients of these functions satisfy |*a*_*n*_ | ≤1  (*n* ≥ 2) [5–7]. The *n*th coefficient of close-to-convex functions with argument *β* satisfies |*a*_*n*_ | ≤1 + (*n* − 1)cos⁡*β* [8], and the coefficients of uniformly starlike functions are bounded by 2/*n* [9], while |*a*_*n*_ | ≤1/*n* [10] for uniformly convex functions. Simple examples show that these bounds are not sufficient to characterize the geometric properties of the classes of functions.

In the sequel, we will assume that *f* ∈ *A* has the Taylor expansion of the form *f*(*z*) = *z* + ∑_*n*=2_^*∞*^ *a*_*n*_ *z* ^*n*^. Gavrilov [11] showed that the radius of univalence for functions *f* ∈ *A* satisfying |*a*_*n*_ | ≤*n*  (*n* ≥ 2) is the real root *r*_0_≃  0.164 of the equation 2(1 − *r*)^3^ − (1 + *r*) = 0, and the result is sharp for *f*(*z*) = 2*z* − *z*/(1 − *z*)^2^. Gavrilov also proved that the radius of univalence for functions *f* ∈ *A* satisfying the coefficient bound |*a*_*n*_ | ≤*M*  (*n* ≥ 2) is $1-\sqrt{M/(1+M)}$. The condition |*a*_*n*_ | ≤*M* clearly holds for functions *f* ∈ *A* satisfying |*f*(*z*)|≤*M*, and for these functions, Landau [12] proved that the radius of univalence is $M-\sqrt{M^{2}-1}$. In fact, Yamashita [13] showed that the radius of univalence obtained by Gavrilov [11] is also the radius of starlikeness for functions *f* ∈ *A* satisfying |*a*_*n*_ | ≤*n* or |*a*_*n*_ | ≤*M*. Additionally, Yamashita [13] determined that the radius of convexity for functions *f* ∈ *A* satisfying |*a*_*n*_ | ≤*n* is the real root *r*_0_≃  0.090 of the equation 2(1 − *r*)^4^ − (1 + 4*r* + *r* ^2^) = 0, while the radius of convexity for functions *f* ∈ *A* satisfying |*a*_*n*_ | ≤*M* is the real root of

(1) $$\begin{array}{c} \left(M+1\right){\left(1-r\right)}^{3}-M\left(1+r\right)=0. \end{array}$$

Recently, Kalaj et al. [14] obtained the radii of univalence, starlikeness, and convexity for harmonic mappings satisfying certain coefficient inequalities.

For two analytic functions *f* and *g*, the function *f* is* subordinate* to *g*, denoted by *f*≺*g*, if there is an analytic self-map *w* of *D* with *w*(0) = 0 satisfying *f*(*z*) = *g*(*w*(*z*)). If *g* is univalent, then *f*≺*g* is equivalent to *f*(0) = *g*(0) and *f*(*D*)⊆*g*(*D*).

For *β* ∈ *R*∖{1},  *α* ≥ 0, the class *L*(*α*, *β*) consists of functions *f* ∈ *A* satisfying

(2) $$\begin{array}{c} \alpha \frac{z^{2}f^{\prime \prime }\left(z\right)}{f\left(z\right)}+\frac{zf^{\prime }\left(z\right)}{f\left(z\right)}≺\frac{1+\left(1-2\beta \right)z}{1-z}. \end{array}$$

Denote by *L*_0_(*α*, *β*) its subclass consisting of functions *f* ∈ *A* satisfying

(3) $$\begin{array}{c} \left|\alpha \frac{z^{2}f^{\prime \prime }\left(z\right)}{f\left(z\right)}+\frac{zf^{\prime }\left(z\right)}{f\left(z\right)}-1\right|\leq \left|1-\beta \right|\;\left(\beta \in R\setminus \left\{1\right\},\alpha \geq 0\right). \end{array}$$

These classes were investigated in [15–24].

For *β* < 1, the class *L*(0, *β*) is the class of starlike functions of order *β*, while, for the case *β* > 1, the class was studied in [25–28].

The class *ST*[*A*, *B*] of Janowski starlike functions [29] consists of *f* ∈ *A* satisfying the subordination

(4) $$\begin{array}{c} \frac{zf^{\prime }\left(z\right)}{f\left(z\right)}≺\frac{1+Az}{1+Bz}\;\left(-1\leq B<A\leq 1\right). \end{array}$$

Certain well-known subclasses of starlike functions are special cases of *ST*[*A*, *B*] for appropriate choices of the parameters *A* and *B*. For example, for 0 ≤ *β* < 1, *ST*(*β*)≔*ST*[1 − 2*β*, −1] is the familiar class of starlike functions of order *β*. Denote by *ST*_*β*_ the class *ST*_*β*_≔*L*_0_(0, *β*) = *ST*[1 − *β*, 0]. Janowski [29] obtained the sharp radius of convexity for *ST*[*A*, *B*].

This paper studies the class *A*_*b*_ consisting of functions *f*(*z*) = *z* + ∑_*n*=2_^*∞*^ *a*_*n*_ *z* ^*n*^,  (|*a*_2_ | = 2*b*,  0 ≤ *b* ≤ 1), in the disk *D*. The subclass of univalent functions in *A*_*b*_ have been studied in [30–33]. In [33], Ravichandran obtained sharp radii of starlikeness and convexity of order *α* for functions *f* ∈ *A*_*b*_ satisfying |*a*_*n*_ | ≤*n* or |*a*_*n*_ | ≤*M*, *n* ≥ 3. The author also obtained the radius of uniform convexity and parabolic starlikeness for functions *f* ∈ *A*_*b*_ satisfying |*a*_*n*_ | ≤*n*, *n* ≥ 3.

This paper finds radius constants for functions *f*(*z*) = *z* + ∑_*n*=2_^*∞*^ *a*_*n*_ *z* ^*n*^ ∈ *A*_*b*_ satisfying either |*a*_*n*_ | ≤*cn* + *d*(*c*, *d* ≥ 0) or |*a*_*n*_ | ≤*c*/*n*  (*c* > 0,  *n* ≥ 3). In the next section, sharp *L*(*α*, *β*)-radius and *ST*[*A*, *B*]-radius are derived for these classes. Several known radius constants are shown to be special cases of the results obtained.

## 2. Radius Constants

A sufficient condition for functions *f* ∈ *A* to belong to the class *L*(*α*, *β*) is given in the following lemma.

**Lemma 1 (see [24, 34]).**

Let *β* ∈ *R*∖{1} and *α* ≥ 0. If *f*(*z*) = *z* + ∑_*n*=2_^*∞*^ *a*_*n*_ *z* ^*n*^ ∈ *A* satisfies the inequality

(5) $$\begin{array}{c} \overset{\infty }{\underset{n=2}{\sum }}\left(\alpha n^{2}+\left(1-\alpha \right)n-\beta \right)\left|a_{n}\right|\leq \left|1-\beta \right|, \end{array}$$

then *f* ∈ *L*(*α*, *β*).

Making use of this lemma, the sharp *L*(*α*, *β*)-radius is obtained for *f* ∈ *A*_*b*_ satisfying the coefficient inequality |*a*_*n*_ | ≤*cn* + *d*.

**Theorem 2 .**

Let *β* ∈ *R*∖{1}, 6*α* + 3 − *β* ≥ 0, and *α* ≥ 0. The *L*(*α*, *β*)-radius for *f*(*z*) = *z* + ∑_*n*=2_^*∞*^ *a*_*n*_ *z* ^*n*^ ∈ *A*_*b*_ satisfying the coefficient inequality |*a*_*n*_ | ≤*cn* + *d*, *c*, *d* ≥ 0, *n* ≥ 3, is the real root in (0,1) of the equation

(6) ((c+d)(1−β)+|1−β| +(2α+2−β)(2(c−b)+d)r)(1−r)4  =cα(1+4r+r2)+((1−α)c+αd)(1−r2)   +((1−α)d−βc)(1−r)2−βd(1−r)3.

For *β* < 1, this number is also the *L*_0_(*α*, *β*)-radius of *f* ∈ *A*_*b*_. The results are sharp.

**Proof**

The number *r*_0_ is the *L*(*α*, *β*)-radius for *f* ∈ *A*_*b*_ if and only if *f*(*r*_0_ *z*)/*r*_0_ ∈ *L*(*α*, *β*). Therefore, by Lemma 1, it is sufficient to verify the inequality

(7) $$\begin{array}{c} \overset{\infty }{\underset{n=2}{\sum }}\left(\alpha n^{2}+\left(1-\alpha \right)n-\beta \right)\left|a_{n}\right|r_{0}^{n-1}\leq \left|1-\beta \right|, \end{array}$$

where *r*_0_ is the real root in (0,1) of (6). Using the known expansions

(8) ∑n=3∞r0n−1=11−r0−1−r0,

(9) ∑n=3∞nr0n−1=1(1−r0)2−1−2r0,

(10) ∑n=3∞n2r0n−1=1+r0(1−r0)3−1−4r0,

(11) ∑n=3∞n3r0n−1=1+4r0+r02(1−r0)4−1−8r0

leads to

(12) ∑n=2∞(αn2+(1−α)n−β)|an|r0n−1 ≤2(2α+2−β)br0  +∑n=3∞(αn2+(1−α)n−β)(cn+d)r0n−1 =2(2α+2−β)br0+cα(1+4r0+r02(1−r0)4−1−8r0)  +((1−α)c+αd)(1+r0(1−r0)3−1−4r0)  +((1−α)d−βc)(1(1−r0)2−1−2r0)  −βd(11−r0−1−r0) =(c+d)(β−1)−(2α+2−β)(2(c−b)+d)r0  +(cα(1+4r0+r02)+((1−α)c+αd)(1−r02)    +((1−α)d−βc)(1−r0)2    −βd(1−r0)3)×(1−r0)−4. =|1−β|.

For *β* < 1, consider the function

(13) f0(z)=z−2bz2−∑n=3∞(cn+d)zn=(c+1)z+2(c−b)z2−cz(1−z)2−dz31−z.

At the root *z* = *r*_0_ in (0,1) of (6), *f*_0_ satisfies

(14) Re(αz2f′′0(z)f0(z)+zf0′(z)f0(z))=1−N(r0)D(r0)=β,

where

(15) N(r0)=−2(c−b)(2α+1)r0+2cr0(2α+1)(1−r0)3 +6cαr02(1−r0)4+2dr02(3α+1)1−r0 +dr03(6α+1)(1−r0)2+2dr04α(1−r0)3,D(r0)=c+1+2(c−b)r0−c(1−r0)2−dr021−r0.

This shows that *r*_0_ is the sharp *L*(*α*, *β*)-radius for *f* ∈ *A*_*b*_. For *β* < 1, (14) shows that the rational expression *N*(*r*_0_)/*D*(*r*_0_) is positive, and therefore the equality

(16) $$\begin{array}{c} \left|\alpha \frac{z^{2}f_{0}^{\prime \prime }\left(z\right)}{f_{0}\left(z\right)}+\frac{zf_{0}^{\prime }\left(z\right)}{f_{0}\left(z\right)}-1\right|=1-\beta \end{array}$$

holds. Thus, *r*_0_ is the sharp *L*_0_(*α*, *β*)-radius for *f* ∈ *A*_*b*_ when *β* < 1.

For *β* > 1, the function

(17) f0(z)=z+2bz2+∑n=3∞(cn+d)zn=(1−c)z+2(b−c)z2+cz(1−z)2+dz31−z

demonstrates sharpness of the result. The derivation is similar to the case *β* < 1 and is omitted.

**Theorem 3 .**

Let *β* ∈ *R*∖{1} and *α* ≥ 0. The *L*(*α*, *β*)-radius of *f*(*z*) = *z* + ∑_*n*=2_^*∞*^ *a*_*n*_ *z* ^*n*^ ∈ *A*_*b*_ satisfying the coefficient inequality |*a*_*n*_ | ≤*c*/*n* for *n* ≥ 3 and *c* > 0 is the real root in (0,1) of the equation

(18) [c(1−β)+|1−β|+(2α+2−β)r(c2−2b)](1−r)2   =cα+(1−α)c(1−r)+βc(1−r)2log⁡(1−r)r.

For *β* < 1, this number is also the *L*_0_(*α*, *β*)-radius of *f* ∈ *A*_*b*_. The results are sharp.

**Proof**

By Lemma 1, *r*_0_ is the *L*(*α*, *β*)-radius of functions *f* ∈ *A*_*b*_ when inequality (7) holds for the real root *r*_0_ of (18) in (0,1). Using (8) and (9) together with

(19) $$\begin{array}{c} \overset{\infty }{\underset{n=3}{\sum }}\frac{r_{0}^{n-1}}{n}=-\frac{\log\left(1-r_{0}\right)}{r_{0}}-1-\frac{r_{0}}{2} \end{array}$$

leads to

(20) ∑n=2∞(αn2+(1−α)n−β)|an|r0n−1 ≤2(2α+2−β)br0  +∑n=3∞(αn2+(1−α)n−β)(cn)r0n−1 =2(2α+2−β)br0+cα(1(1−r0)2−1−2r0)  +(1−α)c(11−r0−1−r0)  −βc(−log⁡⁡(1−r0)r0−1−r02) =c(β−1)+(2α+2−β)r0(2b−c2)  +cαr0+(1−α)c(1−r0)r0+βc(1−r0)2log⁡⁡(1−r0)(1−r0)2r0 =|1−β|.

To verify sharpness for *β* < 1, consider the function

(21) f0(z)=z−2bz2−∑n=3∞cnzn=(1+c)z+(c2−2b)z2+clog⁡(1−z).

At the root *z* = *r*_0_ in (0,1) of (18), *f*_0_ satisfies

(22) Re(αz2f0′′(z)f0(z)+zf0′(z)f0(z)) =1−(−(c2−2b)r0(2α+1)+cr0α(1−r0)2     +c1−r0+clog⁡(1−r0)r0)  ×((1+c)+(c2−2b)r0+clog⁡(1−r0)r0)−1=β.

Thus, *r*_0_ is the sharp *L*(*α*, *β*)-radius for *f* ∈ *A*_*b*_. For *β* < 1, the rational expression in (22) is positive, and therefore

(23) $$\begin{array}{c} \left|\alpha \frac{z^{2}f_{0}^{\prime \prime }\left(z\right)}{f_{0}\left(z\right)}+\frac{zf_{0}^{\prime }\left(z\right)}{f_{0}\left(z\right)}-1\right|=1-\beta , \end{array}$$

which shows that *r*_0_ is the sharp *L*_0_(*α*, *β*)-radius for *f* ∈ *A*_*b*_. For *β* > 1, sharpness of the result is demonstrated by the function *f*_0_ given by

(24) f0(z)=z+2bz2+∑n=3∞cnzn=(1−c)z+(2b−c2)z2−clog⁡(1−z).

**Remark 4 .**

The results obtained above yield the following special cases.

1. For *α* = 0, *β* = 0, *c* = 1, *d* = 0, and 0 ≤ *b* ≤ 1, Theorem 2 yields the radius of starlikeness obtained by Yamashita [13].
2. For *α* = 0, *c* = 1, and *d* = 0, Theorem 2 reduces to Theorem  2.1 in [33, page 3]. When *α* = 0, *c* = 0, and *d* = *M*, Theorem 2 leads to Theorem  2.5 in [33, page 5].
3. For *α* = 0, Theorem 3 yields the radius of starlikeness of order *β* for *f* ∈ *A*_*b*_ obtained by Ravichandran [33, Theorem 2.8].

The following result of Goel and Sohi [35] will be required in our investigation of the class of Janowski starlike functions.

**Lemma 5 (see [35]).**

Let −1 ≤ *B* < *A* ≤ 1. If *f*(*z*) = *z* + ∑_*n*=2_^*∞*^ *a*_*n*_ *z* ^*n*^ ∈ *A* satisfies the inequality

(25) $$\begin{array}{c} \overset{\infty }{\underset{n=2}{\sum }}\left(\left(1-B\right)n-\left(1-A\right)\right)\left|a_{n}\right|\leq A-B, \end{array}$$

then *f* ∈ *ST*[*A*, *B*].

The next result finds the sharp *ST*[*A*, *B*]-radius for *f* ∈ *A*_*b*_ satisfying the coefficient inequality |*a*_*n*_ | ≤*cn* + *d*.

**Theorem 6 .**

Let −1 ≤ *B* < *A* ≤ 1. The *ST*[*A*, *B*]-radius for *f*(*z*) = *z* + ∑_*n*=2_^*∞*^ *a*_*n*_ *z* ^*n*^ ∈ *A*_*b*_ satisfying the coefficient inequality |*a*_*n*_ | ≤*cn* + *d*, *n* ≥ 3 and *c*, *d* ≥ 0, is the real root in (0,1) of the equation

(26) [(A−B)(c+d+1) −(2b−2c−d)(2(1−B)−(1−A))r](1−r)3   =c(1−B)(1+r)+(d(1−B)−c(1−A))(1−r)    −(1−A)d(1−r)2.

This radius is sharp.

**Proof**

It is evident that *r*_0_ is the *ST*[*A*, *B*]-radius of *f* ∈ *A*_*b*_ if and only if *f*(*r*_0_ *z*)/*r*_0_ ∈ *ST*[*A*, *B*]. Hence, by Lemma 5, it suffices to show that

(27) ∑n=2∞((1−B)n−(1−A))|an|r0n−1≤A−B             (−1≤B<A≤1),

where *r*_0_ is the root in (0,1) of (26). From (8), (9), and (10), it follows that

(28) ∑n=2∞((1−B)n−(1−A))|an|r0n−1 ≤2(2(1−B)−(1−A))br0  +∑n=3∞((1−B)n−(1−A))(cn+d)r0n−1 =2(2(1−B)−(1−A))br0  +c(1−B)(1+r0(1−r0)3−1−4r0)  +(d(1−B)−c(1−A))(1(1−r0)2−1−2r0)  −(1−A)d(11−r0−1−r0) =(B−A)(c+d)+(2b−2c−d)  ×(2(1−B)−(1−A))r0  +(c(1−B)(1+r0)    +(d(1−B)−c(1−A))(1−r0)    −(1−A)d(1−r0)2)×(1−r0)−3 =A−B.

The function *f*_0_ given by (13) shows that the result is sharp. Indeed, at the point *z* = *r*_0_ where *r*_0_ is the root in (0,1) of (26), the function *f*_0_ satisfies

(29) |zf0′(z)f0(z)−1|   =(−2(c−b)r0+2dr021−r0+dr03(1−r0)2+2cr0(1−r0)3)    ×(c+1+2(c−b)r0−c(1−r0)2−dr021−r0)−1,|A−Bzf0′(z)f0(z)|  =(c+1)(A−B)+2(c−b)r0(A−2B)c+1+2(c−b)r0−c/(1−r0)2−dr02/(1−r0)   −(c(A−B)(1−r0)2+2cr0B(1−r0)3     −dr02(A−3B)1−r0+dr03B(1−r0)2)   ×(c+1+2(c−b)r0−c(1−r0)2−dr021−r0)−1.

Then, (26) yields

(30) $$\begin{array}{c} \left|\frac{zf_{0}^{\prime }\left(z\right)}{f_{0}\left(z\right)}-1\right|=\left|A-B\frac{zf_{0}^{\prime }\left(z\right)}{f_{0}\left(z\right)}\right|\;\left(-1\leq B<A\leq 1,\;z=r_{0}\right), \end{array}$$

or equivalently *f*_0_ ∈ *ST*[*A*, *B*].

**Theorem 7 .**

Let −1 ≤ *B* < *A* ≤ 1. The *ST*[*A*, *B*]-radius for *f*(*z*) = *z* + ∑_*n*=2_^*∞*^ *a*_*n*_ *z* ^*n*^ ∈ *A*_*b*_ satisfying the coefficient inequality |*a*_*n*_ | ≤*c*/*n*, *n* ≥ 3 and *c* > 0, is the real root in (0,1) of the equation

(31) ((c+1)(A−B)−(2(1−B)−(1−A))r(2b−c2))  ×(1−r) =c(1−B)+c(1−A)(1−r)log⁡⁡(1−r)r.

This radius is sharp.

**Proof**

By Lemma 5, condition (27) assures that *r*_0_ is the *ST*[*A*, *B*]-radius of *f* ∈ *A*_*b*_ where *r*_0_ is the real root of (31). Therefore, using (8) and (19) for *f* ∈ *A*_*b*_ yields

(32) ∑n=2∞((1−B)n−(1−A))|an|r0n−1 ≤2(2(1−B)−(1−A))br0  +∑n=3∞((1−B)n−(1−A))(cn)r0n−1 =2(2(1−B)−(1−A))br0  +c(1−B)(11−r0−1−r0)  −c(1−A)(−log⁡⁡(1−r0)r0−1−r02) =c(B−A)+(2(1−B)−(1−A))r0(2b−c2)  +c(1−B)r0+c(1−A)(1−r0)log⁡⁡(1−r0)(1−r0)r0 =A−B.

The result is sharp for the function *f*_0_ given by (21). Indeed, *f*_0_ satisfies

(33) |zf0′(z)f0(z)−1| =−(c/2−2b)r0+c/(1−r0)+(clog⁡(1−r0))/r0(1+c)+(c/2−2b)r0+(clog⁡(1−r0))/r0,|A−Bzf0′(z)f0(z)| =((1+c)(A−B)+(A−2B)(c2−2b)r0   +cB1−r0+cAlog⁡(1−r0)r0)  ×((1+c)+(c2−2b)r0+clog⁡(1−r0)r0)−1,

at the root *z* = *r*_0_ in (0,1) of (31). Evidently, the function *f*_0_ satisfies (30), and hence the result is sharp.
